# Unmet needs in long-term care and their associated factors among the oldest old in China

**DOI:** 10.1186/s12877-015-0045-9

**Published:** 2015-04-12

**Authors:** Haiyan Zhu

**Affiliations:** Department of Sociology, Virginia Tech, 650 McBryde Hall (0137), 225 Stanger Street, Blacksburg, Virginia 24061 USA

**Keywords:** Unmet needs, Long-term care, Oldest old, China

## Abstract

**Background:**

With a rapidly aging population and a decline in the availability of family caregivers, the number of elders in China who have unmet long-term care needs is increasing. Because unmet needs often have negative consequences, it is increasingly important to identify factors associated with unmet needs. Utilizing Andersen’s behavioral model of health services use, this study examines the roles of predisposing factors (demographics), enabling factors (resources), and need (e.g., illness level) in long-term care among the oldest old in China.

**Methods:**

Data from three waves (2005, 2008, and 2011) of the Chinese Longitudinal Healthy Longevity Survey (CLHLS) were analyzed. Four sequential, logistic regression models were designed to investigate how predisposing factors, enabling factors, and need were associated with unmet needs in long-term care.

**Results:**

Logistic regression analyses reveal that the significant factors for *both* rural and urban residents were economic status, someone other than a family member as the primary caregiver, caregivers’ willingness to provide care, timely medication, self-rated health, and self-rated life satisfaction. Significant factors among only *urban* residents were age, a son/daughter-in-law as the primary caregiver, activities of daily living (ADL) disabilities expectation of access to community-based care services, and optimism. Significant factors among only *rural* residents were gender and cognitive function.

**Conclusions:**

The risk of having unmet needs associated with ADL disabilities in long-term care is largely determined by the oldest old’s economic status and caregivers’ willingness to provide care for both rural and urban residents. Given that the availability of informal caregivers—mainly family members—is declining, it is crucial to provide financial assistance to the oldest old, to increase formal services such as paid home service and community-based care services, and to reduce family caregivers’ burden in order to reduce the unmet needs of the oldest old in China.

## Background

Long-term care (LTC) needs primarily refer to the services and support that individuals may require for medical care or for assistance with the basic activities of daily living (ADLs) such as bathing, eating, and dressing. Unmet or under-met needs occur when LTC is unavailable or is insufficient to meet the needs of an individual [1,2]. In contrast to older adults who receive proper LTC, those who have unmet needs have a lower quality of life [3], greater challenges and vulnerabilities associated with daily living activities [4], more physician and emergency-room visits and more hospitalizations [5], more hospital readmissions [6], increased psychological stress [7], and a higher rate of mortality [8]. These findings suggest that the issue of unmet needs is important in the study of LTC, and that identifying factors associated with unmet needs is particularly crucial in reducing the risk of poor health outcomes.

Previous studies have examined factors associated with unmet needs in LTC [1,3,5,9-13]. These studies have shown that low educational attainment, low income, not having a marital partner, living alone, ADL disabilities, cognitive impairment, and having fewer formal or informal resources available were factors associated with having more unmet LTC needs. These significant factors can be summarized by Andersen’s behavioral model of health services use [14]. In this framework, factors that affect the use of health care services are classified into three broad categories: predisposing factors (demographics), enabling factors (resources), and need (e.g., illness level).

While previous studies have examined the factors described by Andersen’s model as well as other factors, some limitations exist. First, with a few exceptions [1,15], most previous studies were conducted in Western countries. Second, the few studies that have been conducted in mainland China (hereafter China) are very limited in depth and scope; in most cases the studies were not based on nationwide samples (e.g., studies conducted only in Shanghai) [16], and the research design for most of the studies in China did not consider factors from all three of Andersen’s broad categories [1]. Third, the sample size of the oldest old (age 80+), who tend to need the most care, was limited and therefore under-represented in previous studies. Fourth, potential differences in rural and urban areas of China have not been thoroughly examined. Research has shown that factors associated with the prevalence of ADL disabilities differ between rural and urban residents [17]; thus, the unmet needs associated with ADLs may also differ for these two groups in China.

As a transitional developing country, China is facing acute challenges in providing the elderly with LTC services. These challenges have been attributed to the dramatic demographic, socioeconomic, and family structure changes over the last several decades [16,18]. The number of people aged 65 and older is projected to grow from 90 million in 2000 to 300 million in 2050 [19]. It was estimated that, in 2005, there were about 5.7 million (5–6%) Chinese elderly aged 65+ who had at least one difficulty with ADLs and needed LTC; this number could reach 27 million in 2050 [1]. Given these projections, it is imperative to find ways to provide LTC for this large and growing population. Analysis of recent longitudinal data is particularly important to produce results that may affect policies that address the challenges that the traditional family care system faces. Although China has launched some policy initiatives to improve the LTC system, institutionalized care, paid home care, and community-based care services still remain limited [18]. Many of these services cannot provide professional and skilled nursing care [16,18].

The model of elderly care in China is based on the traditional norm of filial piety [20]. In keeping with this tradition, LTC is predominantly provided by family members [1]. However, the availability of potential caregivers is declining due to changing demographic trends, the weakening of traditional values, greater geographic mobility, and improved gender equality [1,16,18,19,21]. First, declines in both mortality and fertility have not only accelerated population aging but also decreased the size of families. Second, Westernization, modernization, and individualization have eroded the core traditional value—filial piety. The impact of these changes has been to shift family structures from the traditional extended family to the nuclear family, decreasing the level of intergenerational support available. Finally, increased geographical mobility means fewer family members live nearby to support elders, and increased participation of women in the labor force means they are less available to be primary caregivers. Both of these changes have further contributed to the decline in the number of available caregivers and have made the informal elderly-care system even more vulnerable.

Rural–urban residency also plays an important role in LTC needs in China, given the substantial disparities in terms of socioeconomic, medical, and care resources between rural and urban areas. The household registration system (*Hukou*), established in 1955, divided the Chinese population into rural and urban sectors, with policies favoring the urban sector [22]. This system not only limited migration from rural to urban areas, but also limited job opportunities, housing opportunities, pension coverage, and access to medical resources for rural residents, thus creating large disparities between rural and urban areas [23]. As a result, rural residents are less likely to be able to afford formal care such as institutionalized care or private care/services [24]. Even if rural residents can afford formal services, availability is limited, and rural residents tend to be more comfortable following the traditional norms of filial piety [1,25]. Therefore, rural residents are more likely than urban residents to rely on family members to provide care, and thus are more likely to experience unmet care needs.

Given the large size and high growth rate of China’s elderly population; the fact that a sizable portion of LTC is provided by family members; the vast urban–rural differences in resources, availability, and accessibility; and the growing numbers of elderly in need of LTC [1,15], it is imperative that we gain a deeper understanding of unmet needs and their associated factors in this transitional country. By using a nationwide survey focusing on the oldest old population and employing Andersen’s framework, the present study aims to investigate: (1) the prevalence of unmet needs among the oldest old in China, (2) the factors associated with unmet needs in LTC, and (3) whether there are differences between urban and rural areas.

## Methods

### Study sample

The study sample is from the three most recent waves (2005, 2008, and 2011) of the Chinese Longitudinal Healthy Longevity Survey (CLHLS). Because no information on unmet needs was collected before the 2005 wave, the first three waves (1998, 2000, and 2002) were not included in our analysis. The CLHLS was initiated in 1998 and approved by the Institutional Review Board at Duke University Medical Center. Informed consent was obtained from each respondent. The CLHLS attempted to interview all centenarians in a randomly selected sample of half of the counties/cities in twenty-two Han-dominated provinces in China [8]. The in-home interviews were carried out by a trained interviewer accompanied by a doctor, nurse, or medical school student who performed a basic health examination for every participant. All respondents in the 2005 wave were re-interviewed in the 2008 and 2011 waves if they were still alive. If they died before the 2008 or 2011 wave, information was collected from the next-of-kin. To replace those who died before the 2008 wave or those who were lost to follow-up due to refusal or migration, the CLHLS recruited new participants of the same age and sex as the drop-outs to ensure the sample would remain comparable to previous waves. The newly recruited participants in 2008 were oversampled in seven longevity areas, and this proportion of respondents accounted for 15% of the total in 2008. However, in 2011, the CLHLS did not recruit new participants due to budget cuts. The response rate in each of these three waves was about 85–90%.

We focused our analysis on the oldest old, respondents who were aged 80–109 at the time of interview and had received assistance in ADLs for at least three months; we disregarded data from respondents older than 109 years in each wave because the data were not reliable [26]. For the purpose of this study, we further excluded those who were institutionalized, which represented only a very small proportion of the sample.

In the 2005 wave, there were 2,938 community-residing respondents age 80+ who had a disability in performing at least one of six ADLs (bathing, dressing, toileting, in-door moving, incontinence, and eating) for at least three months. The corresponding numbers in the 2008 and 2011 waves were 2,919 and 1,647, respectively. To improve the robustness of the results, we pooled these three waves together. A variable indicating the interview wave was included in the analysis in order to control for the potentially confounding effect of different sampling strategies in different waves. Because the surviving respondents in the subsequent waves (i.e., 2008 or 2011) were re-interviewed, they contributed to our analytical sample with multiple observations if they still needed assistance at the time of subsequent interviews. Therefore, strictly speaking, we have 7,504 cases in total from 5,177 individuals. The assessments on accuracy of age-reporting, reliability, validity, and the consistency of numerous measures and the randomness of attrition show that the data quality is high in the CLHLS [26,27].

### Measures

#### Unmet needs

There is no consensus on how to measure LTC needs [1,8]. Following an approach used by some scholars for the U.S. National Long-term Care Survey [28] and for the CLHLS [1,8], we defined LTC need as requiring assistance for more than three months in performing any of the following six ADLs: bathing, dressing, toileting, in-door moving, incontinence, and eating. The CLHLS survey asked if a respondent needed assistance in any of the six tasks. Respondents who answered “yes” were then asked how long they had been receiving assistance. Those who had received ADL assistance for more than 90 days (roughly three months) were included in the study sample. The status of unmet needs was measured based on individuals’ self-reported response to the question, “Does the assistance provided by caregivers meet your needs?” Responses to this question fell into three categories: fully met, partially met, and not met. We combined partially met and not met into one category, following the practice of previous research [1] and because only 3% of the sample reported that needs were not met. This allowed us to dichotomize the responses into two categories: fully met (coded as 0) and unmet (coded as 1). Although this measure of unmet needs has some subjective elements, there is evidence that it is a valid indicator to measure unmet needs [29].

#### Independent variables

Our investigation of factors associated with unmet needs follows a model developed for utilization of and access to LTC service, including predisposing factors, enabling resources, and need [14]. Predisposing factors included demographics–age, gender, and ethnicity (Han versus non-Han). Enabling resources included three subsets of variables: socioeconomic status (SES), caregiving resources, and the availability of community-based care services. Measures of SES included years of schooling of the respondent Measures of SES included years of schooling of the respondent (0, 1–6, 7+), whether primary lifetime occupation was white collar (yes versus no), financial independence (yes versus no), and better perceived economic status of the family compared to most neighbors (yes versus no). Measures of caregiving resources included whether the respondent could get adequate medication when in need (yes versus no), currently married (yes versus no), number of living children, co-residence with children (yes versus no), primary caregiver for ADL assistance (spouse, daughter/son-in-law, son/daughter-in-law, others), and caregivers’ willingness to provide care (yes versus no). A primary caregiver is someone who was actually providing care to the respondent. Care service availability in the neighborhood included whether a program offered personal care services (yes versus no) and whether an individual would expect to have access to such a service (yes versus no).

Need factors included severe ADL disability (i.e., unable to perform at least three of the six ADL tasks) (yes versus no), cognitive impairment (i.e., Mini-Mental Status Examination score lower than 24) [30] (yes versus no), good self-rated health (yes versus no), good self-rated life satisfaction (yes versus no), and having 1+ chronic diseases (yes versus no). As a measure of psychological well-being, optimism (i.e., whether the respondent often looks at the bright side of things) (yes versus no), was also included as a need factor in the analysis.

#### Control variables

To account for the confounding effect of different sampling strategies in the three waves of the CLHLS and for the period effect, we controlled for two variables: whether the respondent was from an area where longevity rates were high (yes versus no), and the particular wave (2005, 2008, 2011). Both variables were controlled for in each of four models in the multivariate analyses.

We further included the frequency of involvement in eight leisure activities, and a dummy variable of whether the participant often does regular exercise (yes versus no). These two variables could reduce ADL disability and may be indirectly correlated to unmet needs. Since the study follows Andersen’s framework [14] and only focuses on predisposing, enabling, and need factors in the analysis, these two variables are treated as control variables and they were only included in the final model in which need factors (i.e., health variables) were present. The frequency of participation in eight leisure activities (relaxing outdoors, gardening, raising pets/domestic poultry, playing cards or mahjong, reading books/newspapers, watching TV/listening to the radio, participating in group-based activities, and doing housework) was measured by five gradient scores for each activity: almost every day (5), at least once per week (4), at least once per month (3), seldom (2), and never (1). We added these scores to generate a frequency of leisure activity index with a total score ranging from 8 to 40.We dichotomized this index into two categories: scored less than or equal to 11 (coded as 0) and scored more than 11 (coded as 1), given the skewed distribution of these scores.

### Statistical analyses

Four sequential models were designed to investigate the association of unmet needs in LTC with respect to each set of factors in terms of predisposing, enabling, and need factors. Model I included demographic variables and socioeconomic variables. Model II added the variables of individual/family caregiving resources. Model III further added the availability of personal care service programs in the neighborhood and expectation to have access to such services. In addition to the variables in the previous models, Model IV included need factors such as health conditions. This modeling strategy was aligned with the health services utilization framework created by Andersen [14]. In addition, we performed all analyses separately for urban and rural areas. As discussed above, there is a vast urban–rural difference in resources. Moreover, our supplementary analysis also revealed a significant difference in unmet needs between urban and rural areas. Binary logistic regressions with random effects were performed in the multivariate analysis to adjust for intrapersonal correlations across waves. No collinearity among variables in the models was found (the largest value of the variance inflation factor was less than 2.5). All statistical analyses were performed using Stata version 12.1.

Missing data were rare in this study. In the worst cases, the missing rate was less than 2%. We used the mode for categorical variables and mean for continuous variables to impute missing values. Multiple imputation methods were also tested and the results were almost identical.

## Results

### Sample characteristics

Table 1 presents the characteristics of the study sample. The proportions of respondents residing in rural and urban areas were about the same: 50.3% vs. 49.7%. More than 40% of respondents were in the 100+ age group in both rural and urban areas (46.1% vs. 43%). Women accounted for more than two thirds of the sample (74% of rural respondents and 66.3% of urban respondents). Overall, the respondents’ SES was low: most of the respondents had less than seven years of schooling and the majority had non-white collar jobs; this was especially true for rural residents. The percentage of those who were currently married was low (10.7% of rural residents vs. 12.8% of urban residents). In both rural and urban areas, most of the respondents co-resided with their children. Children, especially sons/daughters-in-law, were identified as the primary caregivers; this indicates that family care is still the predominant care format in China. However, a noticeable difference between rural and urban residents was that 67% of the rural oldest old rely on sons/daughters-in-laws for care, but this proportion was about 20% lower in urban areas. In terms of health conditions, rural oldest old were more likely to report poor self-rated health and cognitive impairment but less likely to have one or more chronic diseases than their urban counterparts.

**Table 1:** **Sample distribution by rural/urban status**

	**Rural (n = 3,774)**	**Urban (n = 3,730)**		**Rural (n = 3,774)**	**Urban (n = 3,730)**
Total sample (%)	50.3^a^	49.7^a^	Primary caregiver (%)		
Age groups (%)			Spouse	5.4	6.3
80-89 years	16.4	18.9	Son/daughter-in-law	67.3	46.4
90-99 years	37.5	38.1	Daughter/son-in-law	12.7	21.9
100+ years	46.1	43.0	Other persons	14.6	25.5
Sex (%)			Caregiver is willing to provide care (%)		
Women	74.0	66.3	No	14.0	12.9
Men	26.0	33.7	Yes	86.0	88.1
Ethnicity (%)			Community personal care service is available (%)		
Non-Han	6.3	4.4	No	98.2	94.4
Han	93.7	95.6	Yes	1.8	5.6
Wave (%)			Expectation of access to community-based care services (%)		
2005	36.7	41.6	No	38.1	44.0
2008	42.4	35.4	Yes	61.9	56.0
2011	21.0	23.0	Average score of frequent leisure activities (ranges 8–40)	10.5	11.8
Years of schooling (%)			Doing regular exercise (%)		
0	83.4	67.6	No	90.8	83.3
1-6	13.8	23.1	Yes	9.2	16.7
7+	2.8	9.3	Severely ADL disabled (%)		
White collar job (%)			No	63.5	63.5
No	97.6	90.2	Yes	36.5	36.5
Yes	2.4	9.8	Cognitively impaired (%)		
Financial independence (%)			No	16.3	27.2
No	25.7	22.4	Yes	83.7	72.8
Yes	74.3	77.6	Good self-rated life satisfaction (%)		
Family is rich compared to neighbors (%)			No	58.2	49.2
No	87.0	83.0	Yes	41.8	50.8
Yes	13.0	17.0	Good self-rated health (%)		
Timely medication (%)			No	72.5	70.1
No	13.0	6.0	Yes	27.5	29.9
Yes	87.0	94.0	Optimistic (%)		
Currently married (%)			No	54.9	45.5
No	89.3	87.2	Yes	45.1	54.5
Yes	10.7	12.8	Having 1+ chronic diseases (%)		
Average no. of living children	3.2	3.4	No	39.0	27.6
Coresidence with children (%)			Yes	61.0	72.4
No	11.9	11.6	Living in longevity areas (%)		
Yes	88.1	88.4	No	90.4	92.2
			Yes	9.6	7.8

Note: (1) The results are unweighted. (2) ^a^,% is calculated in all samples. All other % or means are calculated for urban and rural areas separately.

### Prevalence of unmet needs

Table 2 reveals the prevalence of unmet needs for both rural and urban oldest old by year. Rural residents had a higher prevalence of unmet needs compared to their urban counterparts across all three waves. The prevalence of unmet needs in all three years was similar among urban residents, between about 53% and 54%, which suggests little change between 2005 and 2011 among urban residents. By contrast, the prevalence of unmet needs was more variable in rural areas, ranging from about 56.5% to 65%. The largest difference in the prevalence of unmet needs between rural and urban residents was found in the 2005 wave (64.7% vs. 53.5%).

**Table 2:** **Prevalence of unmet LTC needs, 2005–2011 (n = 3,774 for rural; n = 3,730 for urban)**

**Unmet needs**	**2005**		**2008**		**2011**	
	**Rural**	**Urban**	**Rural**	**Urban**	**Rural**	**Urban**
No	489 (35.3%)	723 (46.5%)	696 (43.5%)	607 (46%)	320 (40.5%)	401 (46.9%)
Yes	895 (64.7%)	831 (53.5%)	903 (56.5%)	713 (54%)	471 (59.5%)	455 (53.1%)
Total	1,384	1,554	1,599	1,320	791	856

### Multivariate results

#### Rural

Table 3 presents the odds ratios of unmet needs with various sets of factors included among rural residents. Gender was the only significant demographic factor associated with unmet needs, with men having greater odds of unmet needs than women in all four models. Both economic factors were significantly associated with unmet LTC needs in all four models: financial independence reduced the odds of having unmet needs by 57–71% and better economic status of family relative to neighbors reduced the odds by 48–60%. Additional analysis of enabling factors shows that receiving timely medication and having a caregiver who was willing to provide care were associated with decreased odds of unmet needs (Models II-IV). In Model IV, with all other factors controlled for, caregivers’ willingness had the greatest impact on unmet needs. If a caregiver was willing to provide care, the odds of having unmet needs decreased by 78%. If the caregiver was someone other than the respondent’s spouse or son/daughter-in-law (compared with the respondent’s daughter/son-in-law), the odds of having unmet needs was 39% higher. Regarding need factors, good self-rated life satisfaction and good self-rated health reduced the odds of having unmet needs by 30% and 43%, respectively, while cognitive impairment increased the odds of having unmet needs by 29%, as shown in Model IV. In addition, a control variable, doing regular exercise, reduced the odds of having unmet needs by 35%.

**Table 3:** **Odds ratios of factors associated with unmet LTC needs, 2005–2011, rural (n = 3,774)**

	**Models**			
	**I**	**II**	**III**	**IV**
Age	1.00	1.01	1.01	1.00
Men (women)	1.34**	1.33*	1.34*	1.45**
Han ethnicity (non-Han)	0.97	1.03	1.04	1.08
2008 wave (2005)	0.64***	0.70***	0.70***	0.62 ***
2011 wave (2005)	0.82	0.84	0.83	0.79
Longevity areas (no)	0.86	0.89	0.89	0.97
1-6 years of schooling (0)	0.84	0.80	0.80	0.84
7+ years of schooling (0)	0.80	0.84	0.82	0.87
White collar job (no)	1.40	1.30	1.31	1.32
Financial independence (no)	0.29***	0.36***	0.36***	0.43***
Family is rich compared to neighbors (no)	0.40***	0.43***	0.43***	0.52***
Timely medication (no)		0.47***	0.47***	0.56***
Currently married (no)		1.48*	1.49*	1.44
Number of living children		0.96	0.96	0.97
Coresidence with children (no)		0.81	0.81	0.84
Spouse is the primary caregiver (daughter/son-in-law)		0.69	0.69	0.66
Son/daughter-in-law is the primary caregiver (daughter/son-in-law)		1.18	1.19	1.20
Other person is the primary caregiver (daughter/son-in-law)		1.33	1.35	1.39*
Caregiver is willing to provide care (no)		0.20***	0.20***	0.22***
Care service is available in neighborhood (no)			0.52*	0.61
Expectation of access to neighborhood care service (no)			1.15	1.07
Severe ADL disability (no)				1.17
Cognitively impaired (no)				1.29*
Good self-rated life satisfaction (no)				0.70***
Good self-rated health (no)				0.57***
Optimistic (no)				0.95
Having 1+ chronic diseases (no)				0.99
Frequent involvement in leisure activities				0.82
Doing regular exercise (no)				0.65**
rho	0.164*	0.170*	0.174*	0.168*
Wald chi square	126.4***	144.7***	145.1***	153.2***

Note: (1) Odds ratios are based on the logistic regressions adjusting for intrapersonal correlations. (2) Category in the parentheses of a categorical or dummy variable is the reference category. (3) *p < 0.05, **p < 0.01, ***p < 0.001.

#### Urban

Table 4 replicates the analysis in Table 3 for urban residents. Predisposing demographic factors were different for urban residents than for rural residents. Instead of gender, age was the only significant demographic factor: each additional year of age was associated with 1-2% lower odds of having unmet needs across all four models. The effects of SES factors on urban residents’ risk of unmet needs were similar to the effects on rural residents’ risk: financial independence and better family economic conditions were negatively associated with the risk of having unmet needs. Receiving timely medication and having a caregiver who was willing to provide care were also associated with lower risks of having unmet needs among urban residents in all four models. For example, financial independence reduced the risk of having unmet needs by 35%, but caregivers’ willingness to provide care remained the most influential factor: Having a caregiver who was willing to provide services reduced the risk of having unmet needs by 77% (Model IV). As with the rural oldest old, having someone other than a family member increased the urban oldest old’s odds of having unmet needs by 47%, compared with having a daughter/son-in-law as the primary caregiver. Unlike the results among rural residents, a significant difference was found between sons/daughters-in-law and daughters/sons-in-law as the primary caregivers; having sons/daughters-in-law as the primary caregivers (instead of daughters/sons-in-law) increased the odds of having unmet needs by 35% for urban respondents. Moreover, expected access to neighborhood care services (vs. no expectation) was associated with 30% increased odds of having unmet needs (Model IV). Furthermore, need measured as severe ADL disability, increased the odds of having unmet needs by 21%; good self-rated health and good self-rated life satisfaction reduced the odds of having unmet needs by 25% and 33%, respectively. Finally, for urban residents, optimism was associated with 30% lower odds of having unmet needs.

**Table 4:** **Odds ratios of factors associated with unmet LTC needs, 2005–2011, urban (n = 3,730)**

	**Models**			
	**I**	**II**	**III**	**IV**
Age	0.99*	0.99*	0.99*	0.98*
Men (women)	0.89	0.85	0.86	0.92
Han ethnicity (non-Han)	0.83	0.83	0.81	0.79
2008 wave (2005)	1.01	1.00	0.98	0.94
2011 wave (2005)	1.01	1.00	0.97	0.95
Longevity areas (no)	1.07	1.08	1.06	0.99
1-6 years of schooling (0)	1.08	1.11	1.10	1.14
7+ years of schooling (0)	1.34	1.35	1.35	1.38
White collar job (no)	0.88	0.86	0.86	0.89
Financial independence (no)	0.48***	0.56***	0.57***	0.65***
Family is rich compared to neighbors (no)	0.49***	0.48***	0.49***	0.56***
Timely medication (no)		0.58**	0.59***	0.67*
Currently married (no)		1.14	1.14	1.12
Number of living children		0.98	0.98	0.99
Coresidence with children (no)		1.00	1.02	1.02
Spouse is the primary caregiver (daughter/son-in-law)		1.08	1.07	1.03
Son/daughter-in-law is the primary caregiver (daughter/son-in-law)		1.37**	1.35**	1.35**
Other person is the primary caregiver (daughter/son-in-law)		1.54***	1.49***	1.47**
Caregiver is willing to provide care (no)		0.19***	0.19***	0.23***
Care service is available in neighborhood (no)			1.27	1.35
Expectation of access to neighborhood care service (no)			1.34***	1.30**
Severe ADL disability (no)				1.21*
Cognitively impaired (no)				1.20
Good self-rated life satisfaction (no)				0.67**
Good self-rated health (no)				0.75**
Optimistic (no)				0.70***
Having 1+ chronic diseases (no)				0.97
Frequent involvement in leisure activities				0.93
Doing regular exercise (no)				0.80
rho	0.058	0.059	0.057	0.061
Wald chi square	113.9***	177.1***	179.5***	217.8***

Note: (1) Odds ratios are based on the logistic regressions adjusting for intrapersonal correlations. (2) Category in the parentheses of a categorical or dummy variable is the reference category. (3) *p < 0.05, **p < 0.01, ***p < 0.001.

## Discussion

Based on the 2005, 2008, and 2011 waves of the CLHLS, this study examined unmet needs in LTC and associated factors among the oldest old Chinese. The significant factors associated with unmet needs can be divided into three categories based on patterns for rural and urban residents: (1) factors significant for both rural and urban residents: economic status, timely medication, someone other than a family member as the primary caregiver, caregivers’ willingness to provide care, self-rated health, and self-rated life satisfaction, (2) factors significant only for rural residents: gender and cognitive impairment, and (3) factors significant only for urban residents: age, a son/daughter-in-law as the primary caregiver, expectation of access to community-based service, severe ADL disability, and optimism. We will highlight and discuss some important factors in each of these three categories below.

### Common factors

Our results show that caregivers’ willingness, type of primary caregiver, and economic status were especially important for both rural and urban residents. Caregivers’ willingness to provide care was the most influential factor associated with unmet needs, a finding that is in line with previous studies [15]. Clearly, caregivers’ willingness to provide care can influence care quality and thus indirectly impact the unmet needs of the oldest old. Receiving care from unwilling caregivers may make care recipients feel less satisfied with the care provided, and thus more likely to report unmet needs. The risk of having unmet needs was also higher if someone other than a family member was the primary caregiver (compared with a daughter/son-in-law as the primary caregiver). This is understandable because familiarity and trust are necessary for the oldest old to accept assistance in personal ADLs such as bathing and toileting [15]. Further, because there is a lack of professional, skilled nursing care services in both rural and urban China [16,18], family members are still likely the best available care providers. Economic status was associated with both rural and urban residents’ risk of having unmet needs, which is consistent with previous studies [3,5,9,15]. Oldest old who are more financially secure generally have greater access to medical resources and are better able to pay for health care services, thus lowering their risk of having unmet needs.

### Rural factors

Our findings show that being male and cognitively impaired were associated with more unmet needs among rural but not urban oldest old. Consistent with previous literature [15], in rural areas men had a higher risk of having unmet needs than women. Research has found that women tend to receive fewer hours of care than men and are more likely to be caregivers even when they experience their own disability [31]; this can be explained by sociocultural patterns such as the lower engagement of women in paid employment and the traditional association of women with caregiving roles [31,32]. This pattern is particularly strong in rural China. It is possible that rural women reported less unmet need because they had lower expectations for care and thus were more likely to be satisfied with the care they received.

We speculate that several factors that were not included in the analysis may explain why cognitive impairment was significant for rural but not for urban residents. First, diagnosis and treatment of cognitive impairment may be neglected in rural areas due to poor health literacy and limited access to medical services. Second, previous studies have suggested that lower caregiver education level is associated with lower quality of care [33], thus increasing the likelihood of care recipients having unmet needs. Rural caregivers’ lower education [34] may correlate with lack of caregiving knowledge, which might explain why there is a significant relationship between cognitive impairment and the risk of having unmet needs in rural areas.

### Urban factors

The study found that several factors were associated with the risk of having unmet needs only for urban residents. These factors were type of primary caregiver, ADL disability, age, and optimism. Having a son/daughter-in-law as the primary caregiver was associated with greater odds of unmet needs compared to having a daughter/son-in-law as the primary caregiver in urban areas; in other words, daughters/sons-in-law provided better care than sons/daughters-in-law. A number of reasons could explain the advantage of daughters/sons-in-law as the primary caregivers in urban areas. Due to traditional divisions of labor and the nature of caregiving, women tend to take on more caregiving responsibilities [31], and they also tend to be better caregivers than men [35,36]. Despite the fact that sons’ wives are women, parents tend to have a closer emotional tie with daughters than daughters-in-law [36]; as a result, daughters are more likely to provide good care and parents are more likely to be satisfied with the care daughters provide. In contrast, the tension between daughters-in-law and mothers-in-law is the most frequent dispute in intergenerational relationships in China [37]. Although daughters are predominately regarded as better caregivers, this study did not find that having a daughter as the primary caregiver was associated with a lower likelihood of having unmet needs in rural areas compared with having a son/daughter-in-law as the primary caregiver. This may be due to the rural oldest old’s suppression of needs [38], because they may be used to hard living conditions and do not have or have limited access to community-based care services; thus, they are more likely to be satisfied with lower levels of care and make less of a distinction between the care they receive from a daughter/son-in-law or a son/daughter-in-law. In contrast, the urban oldest old tend to have higher expectations given their higher standards of living and thus are more likely to be satisfied with caregiving by a daughter.

Moreover, severe ADL disability was associated with a higher risk of unmet needs among urban residents but not rural residents. Supplementary data analyses (not shown here) revealed that the bivariate association between severe ADL disability and unmet needs was explained away by other health conditions and by regular exercise, especially the latter, in rural areas, but remained significant independent of regular exercise and other health conditions in urban areas. Further analysis showed that rural residents who regularly exercised were less likely to experience severe ADL disability compared with urban residents who regularly exercised. This study was not able to investigate why regular exercise would benefit rural residents more than urban residents because we lacked data on frequency, duration, and history of exercising. These uncontrolled factors should be considered in future studies.

Finally, age and optimism were both related to unmet needs for the urban oldest old. In contrast to previous studies [1,15], we found that older age was associated with a slightly lower risk of having unmet needs compared to younger age in urban areas. Older old residents, who may be less able to engage in daily activities, may require less assistance than younger old residents in urban areas. As discussed above, suppression of care needs and expectations in rural areas may explain why there was no age difference in unmet needs among rural oldest old. Likewise, optimism may be important for urban oldest old because they tend to have higher expectations for quality of life due to higher standards of living and community-based care services. In contrast, rural residents’ suppression of needs and lower expectations for receiving care likely negate the effects of optimism.

### Limitations

The study has several limitations. First, availability of community-based care services is the only factor at the community level included in the study. Andersen and Davidson suggest that predisposing, enabling, and need factors should be measured at the community context level as well as the individual level in the framework of health care utilization [39]. For example, lack of care resources in an area increases the risk for unmet needs among the elderly; if the dependency ratio of elders is high in an area, then the care resources in that area may be insufficient, resulting in more unmet needs. Therefore, given substantial regional variations in demographics, SES, medical resources, and health care resources in China, community-level variables related to demographics (e.g., proportion of elders with disabilities and dependency ratio of elders), SES (e.g., local government budgets for care services), and care resources (e.g., number of home service workers) should be included in future studies [12].

Second, this study lacked data on care-resource factors such as the number of hours of care received and primary caregivers’ characteristics such as age and education. The effect of a caregiver’s age on the risk of having unmet needs is stronger in the case of the oldest old because their caregivers, mostly spouses or children, are also old or very old and may also need assistance with ADLs. Additionally, people with caregivers who have low levels of education are more likely to have low quality of care [33], likely due to lack of knowledge and competence. Future studies should also include these variables as factors associated with unmet needs, especially when focusing on rural–urban differences.

Third, this study analyzed pooled data from the 2005, 2008, and 2011 waves of CLHLS. Given the rapid speed of urbanization in contemporary China, it is possible that some rural areas in 2005 had been transformed into urban areas by 2011. This possible change in the classification of residences may introduce a bias to the estimate of unmet needs in rural and urban areas. However, we do not think the major results are affected by this bias because the change in the classification of residences should not be large within a six-year period.

## Conclusion

Many predisposing, enabling, and need factors in the Andersen model were significantly associated with unmet LTC needs among the oldest old in China. Economic status and caregivers’ willingness to provide care were the most important enabling factors for both rural and urban oldest old. Predisposing factors such as age and gender, enabling factors such as type of primary caregiver, and need factors such as ADL disability and cognitive impairment differed between urban and rural oldest old, which may be mostly explained by differing expectations for care which stem from differences in their standards of living and access to care resources.

These findings have important implications for policy makers. Since economic status plays an important role in shaping unmet needs among the oldest old, providing financial assistance or some insurance coverage would be an effective way to help elders meet their needs. Improved economic conditions would enable the elderly to have access to medical services, including timely medical treatment, and to more care resources such as paid home care, thus decreasing their unmet needs. This assistance is particularly important for rural residents as they have lower SES and a higher prevalence of unmet needs.

Given the decline in both willingness and availability of caregivers, it is imperative to increase the development of formal care services such as paid home-based care and community-based care services. Currently available formal care accounts for less than 10% of all LTC in China [1]; moreover, the cost of formal care is unaffordable for most elders [1,24]. Therefore, formal care has not been able to replace or supplement family support [1,18]. As this study shows, willingness of caregivers is the strongest risk factor for unmet needs among the oldest old. Providing care for people at very old ages—the group who need the most care given their poor relative economic and health status compared to the younger old—can exert heavy physical, psychological, and financial burden on caregivers. Care burden can not only negatively affect caregivers’ quality of life and attitudes toward providing assistance, but also discourage potential caregivers from being willing to provide care. Nevertheless, although the willingness of caregivers to provide care is declining, family care will probably still prevail for the near future. Given the influence of filial piety, families may be reluctant to place their old family members in an institution where care is provided by strangers [18]. Developing home-based and community-based services will help reduce the burden on informal caregivers while also contributing to the construction of a long-term care system.
